# Hematological and biochemical investigations on the effect of curcumin and Thymoquinone in male mice exposed to Thioacetamide

**DOI:** 10.1016/j.sjbs.2021.10.037

**Published:** 2021-10-22

**Authors:** Atef M. Al-Attar

**Affiliations:** Princess Dr. Najla Bint Saud Al-Saud Center for Excellence Research in Biotechnology, King Abdulaziz University, Jeddah, Saudi Arabia; Department of Biological Sciences, Faculty of Sciences, King Abdulaziz University, Jeddah, Saudi Arabia

**Keywords:** Thioacetamide, Curcumin, Thymoquinone, Antioxidant, Blood, Mice

## Abstract

Currently, living organisms are increasingly exposed to many toxic chemicals in the environment. These substances pose a threat to human life, other living organisms and ecosystem. In fact, there is an increasing requirement to search for safe therapeutic sources today. Medicinal plants and natural products have become of great importance globally because of their therapeutic potential and medicinal properties, as well as their availability and the absence of harmful side effects for most of them. The present study was designed to explore the potential protective effect of curcumin (CUR) and thymoquinone (TQ) in male rats exposed to thioacetamide (TAA). The experimental mice were divided into eight groups. Group 1 was served as control. Group 2 was exposed to 50 mg/ kg body weight of TAA. Group 3 was exposed to CUR and TAA. Mice of group 4 were treated with TQ and TAA. Mice of group 5 were exposed to CUR plus TQ and TAA. Group 6 was supplemented with CUR. Group 7 was subjected to TQ. Mice of group 8 were treated with CUR and TQ. Hematological and biochemical alterations were evaluated after one month. Significant increases of white blood corpuscles (WBC), alanine aminotransferase (ALT), aspartate aminotransferase (AST), alkaline phosphatase (ALP), total bilirubin (TB), tumor necrosis factor-α (TNF-α) and interleukin-6 (IL-6) values were observed in group 2, while the values of red blood corpuscles (RBC), hemoglobin (Hb(, hematocrit (Hct), glutathione (GSH) and superoxide dismutase (SOD) were statistically decreased. Treatment with CUR, TQ and their combination inhibited the hematological and biochemical alterations induced by TAA toxicity. Moreover, the most protective effect was observed in mice treated with CUR plus TQ. These new results suggested that the protective effect of CUR and TQ attributed to their antioxidant properties.

## 1 Introduction

Globally, living organisms and ecosystems are continually exposed to a very complex mixture of chemicals. Hazardous substances are used in many workplaces today. The toxicity of a substance depends on three factors: its chemical structure, the extent to which the substance is absorbed by the body, and the body's ability to detoxify the substance and eliminate it from the body. Toxic substances can be defined as broad group of chemicals capable of causing harm to living organisms. Human activities have an adverse effect on the environment through the increase of pollution rate (Manisalidis et al., 2020, Weng, 2021). Thioacetamide (TAA, CH_3_CSNH_2_) is used as an antifungal agent and recognized as an experimental toxin. TAA has been used extensively in the development of suitable animal models of acute and chronic liver injury employing various doses, times and routes of its administration. TAA gets metabolized to thioacetamide-S-oxide and acetamide immediately after administration to rats. Thioacetamide-S-oxide binds to macromolecules in a cell that are responsible for the change in cell permeability and Ca^++^ uptake. This interruption of calcium stores increases nuclear volume, enlarges nucleoli, and inhibits mitochondrial activity eventually leading to hepatic necrosis (Bruck et al., 2004, Hajovsky et al., 2012). Generation of a large amount of reactive oxygen species (ROS) due to TAA can overwhelm the antioxidant defense mechanism and damage cellular ingredients such as lipids, proteins, and DNA; this in turn can impair cellular structure and function (Ansil et al., 2011). It has been evaluated that the administration of TAA leads to the cell death by necrosis as well as apoptosis in experimental animals (Chen et al., 2006). Moreover, The effects of TAA are not limited to the liver as profound structural and functional changes have been described in the kidney, testis, thymus, lung, spleen and the intestine (Caballero et al., 2001, Latha et al., 2003, Celik et al., 2016, Ghosh et al., 2016, Schyman et al., 2018).

Medicinal plants have been used from ancient times for the treatment of a large variety of diseases. Recently, a large number of natural products and dietary component have been evaluated as potential chemopreventive agent. Additionally, low patient satisfaction from the consumption of synthetic drugs, due to high costs and side effects of these medications caused an increased tendency to traditional treatments (Al-Attar and Shawush, 2015). Herbal usage to treat a massive spectrum of diseases is developing rapidly. In recent studies special attention has been paid to the protective effects of antioxidants by natural origin compounds against poisoning caused by chemical agents. Recent studies have focused on natural antioxidants owing to their protective effects against the toxicity of various pollutants and pathogenic factors (Abdel-Wahhab et al., 2011, Danaei et al., 2019).

Thymoquinone (TQ) is an active ingredient in *Nigella sativa* Linn, which is traditionally known in Middle Eastern countries as Black seed. *N. sativa* seeds consist of 36–38% fixed oil, protein, alkaloids, and saponin. In addition, 0.4–0.45% of *N. sativa* seeds is essential oil, which is characterized by its major constituent, TQ. Thymoquinone, a monoterpene molecule is chemically known as 2-methyl-5-isopropyl-1, 4-benzoquinone. It is abundantly present in seeds of *N. sativa* L. that is popularly known as black cumin or black seed and belongs to the family *Ranunculaceae* (Ali and Blunden, 2003, Majdalawieh et al., 2017). A review paper on therapeutic potentials of TQ showed that TQ has beneficial medicinal effects in various areas (Gholamnezhad et al., 2015, Kassab and El-Hennawy, 2017, Pehlivan et al., 2020, Alkis et al., 2021). *Curcuma longa* (turmeric) is a perennial member of the *Zingiberaceae* family and is cultivated in India and other parts of Southeast Asia (Perrone et al., 2015). Curcumin (CUR) is a polyphenolic flavonoid and a major coloring agent extracted from *C. longa*. CUR is the most active form of the three different curcumoid extracts in the roots of plants (Du et al., 2012, Akbik et al., 2014). CUR is known has lots of pharmacological properties (Purushothaman and Kuttan, 2017, Takhtfooladi and Takhtfooladi, 2019, Ali et al., 2020, Saied et al., 2021). The main goal of the present study was to investigate the potential protective effects of TQ and CUR against the toxicity of TAA in male mice.

## 2 Material and methods

### 2.1 Animals

One hundred and twenty adult male albino mice of MF1 strain weighing 28.4–32.3 g were used in the present study. Mice were acclimatized to the laboratory conditions for one week prior to the initiation of experimental treatments. The animals were housed in standard cages and maintained in controlled temperature (20 ± 1 °C), humidity (65%) and a 12 h dark-light cycle, with balanced food and free access to water. The principles of laboratory animal care were followed through out the duration of experiment and instruction given by King Abdulaziz University ethical committee was followed regarding experimental treatments.

### 2.2 Experimental design

Mice were distributed into eight groups, each group consisting of 15 animals. The experimental groups were treated as follows:

1. Mice were untreated and served as controls.
2. Mice were given 50 mg/kg body weight of TAA (Sigma-Aldrich Corp., St. Louis, MO, USA) by intraperitoneal injection, daily for one month.
3. Mice were intraperitoneally injected with TAA at the same dose given to group 2 and were orally supplemented with CUR at a dose of 100 mg/ kg body weight/ daily for one month.
4. Mice were intraperitoneally injected with TAA at the same dose given to group 2 and were orally supplemented with TQ at a dose of 100 mg/ kg body weight/ daily for one month.
5. Mice were intraperitoneally injected with TAA at the same dose given to group 2 and were orally supplemented with CUR at a dose of 50 mg/ kg body weight and TQ at a dose of 50 mg/ kg body weight/ daily for one month.
6. Mice were orally supplemented with CUR at a dose of 100 mg/ kg body weight/ daily for one month.
7. Mice were orally supplemented with TQ at a dose of 100 mg/ kg body weight/ daily for one month.
8. Mice were orally supplemented with CUR at a dose of 50 mg/ kg body weight and TQ at a dose of 50 mg/ kg body weight/ daily for one month.

### 2.3 Blood analysis

At the end of experimental period, food was withdrawn from the mice and they were fasted for 8 h but had free access to water and then anaesthetized with diethyl ether. Blood samples were collected from orbital venous plexus in two types of tubes. The first tubes contained calcium EDTA for the measurement of red blood corpuscles count (RBC), hemoglobin (Hb) concentration, hematocrit (Hct) level and white blood corpuscles count (WBC) using auto hematology analyzer (BC-2800). Blood samples in the other tubes were left for a short time to allow clotting. Clear sera were obtained by centrifugation at 2500 rpm for 15 min and then stored at −80 °C. Serum levels of alanine aminotransferase (ALT), aspartate aminotransferase (AST), alkaline phosphatase (ALP) and total bilirubin (TB) were estimated using Dimension Vista® 1500 System, USA. The levels of serum glutathione (GSH) and superoxide dismutase (SOD) were measured using assay kits following the manufacturer’s instructions. Moreover, the levels of tumor necrosis factor-α (TNF-α) and interleukin-6 (IL-6) were measured using enzyme-linked immunosorbent assay (ELISA) kit according to the manufacturer’s instructions.

### 2.4 Statistical analysis

The experimental data were expressed as the mean ± standard deviation (SD) and analyzed using Statistical Package for Social Sciences (SPSS) for Windows version 22.0 software. Statistical significance among the groups was analyzed by one-way analysis of variance followed by Tukey’s multiple comparisons test. *P* ≤ 0.05 was considered statistically significant.

## 3 Results

**Fig. 1:**
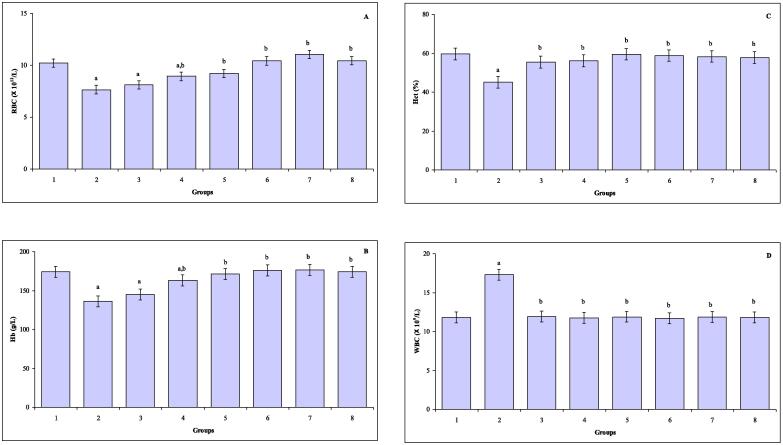
(**A–D**) The values of blood RBC (A), Hb (B), HCT (C) and WBC (D) in control (group 1), TAA (group 2), CUR plus TAA (group 3), TQ plus TAA (group 4), CUR and TQ plus TAA (group 5), CUR (group 6), TQ (group 7) and CUR plus TQ (group 8) treated mice. ^a^Indicates a significant difference between group 1 and treated groups (2, 3, 4, 5,6, 7 and 8). ^b^Indicates a significant difference between group 2, and groups 3, 4, 5, 6, 7 and 8.

The values of blood RBC, Hb, Hct, and WBC, in control, TAA, CUR plus TAA, TQ plus TAA, CUR and TQ plus TAA, CUR, TQ and CUR plus TQ treated mice are represented in Fig. 1A-D. In comparison with control value, the values of RBC were statistically decreased in mice treated with TAA (*P* ≤ 0.001), CUR plus TAA (*P* ≤ 0.001), TQ plus TAA (*P* ≤ 0.01). Significant declines of Hb values were observed in mice exposed to TAA (*P* ≤ 0.000) and CUR plus TAA (*P* ≤ 0.004). The value of Hct (*P* ≤ 0.002) was declined and an increase of WBC value (*P* ≤ 0.000) was noted in TAA intoxicated mice (group 2) compared to control group.

**Fig. 2:**
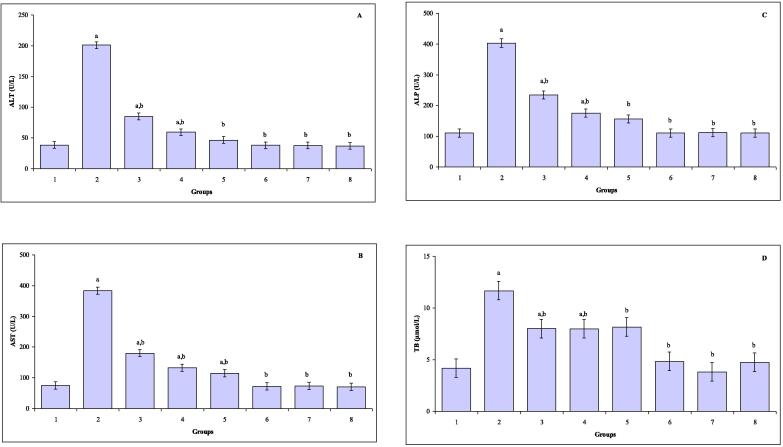
(**A–D**) The levels of serum ALT (A), AST (B), ALP (C) and TB (D) in control (group 1), TAA (group 2), CUR plus TAA (group 3), TQ plus TAA (group 4), CUR and TQ plus TAA (group 5), CUR (group 6), TQ (group 7) and CUR plus TQ (group 8) treated mice. ^a^Indicates a significant difference between group 1 and treated groups (2, 3, 4, 5, 6, 7 and 8). ^b^Indicates a significant difference between group 2, and groups 3, 4, 5, 6, 7 and 8.

The levels of serum ALT, AST, ALP, and TB in all groups are represented in Fig. 2A-D. Significant increases in the level of serum ALT were observed in mice exposed to TAA (*P* ≤ 0.000), CUR plus TAA (*P* ≤ 0.000) and TQ plus TAA (*P* ≤ 0.002). Serum AST levels were significantly increased in mice exposed to TAA (*P* ≤ 0.000), CUR plus TAA (*P* ≤ 0.000), TQ plus TAA (*P* ≤ 0.000) and CUR and TQ plus TAA (*P* ≤ 0.002) compared with control group. Significant elevations in the levels of serum ALP were noted in mice exposed to TAA (*P* ≤ 0.000), CUR plus TAA (*P* ≤ 0.000), TQ plus TAA (*P* ≤ 0.001) and CUR and TQ plus TAA (*P* ≤ 0.02). In comparison with control mice, the levels of serum TB were statistically enhanced in mice treated with TAA (*P* ≤ 0.000), CUR plus TAA (*P* ≤ 0.004), TQ plus TAA (*P* ≤ 0.001) and CUR and TQ plus TAA (*P* ≤ 0.000).

**Fig. 3:**
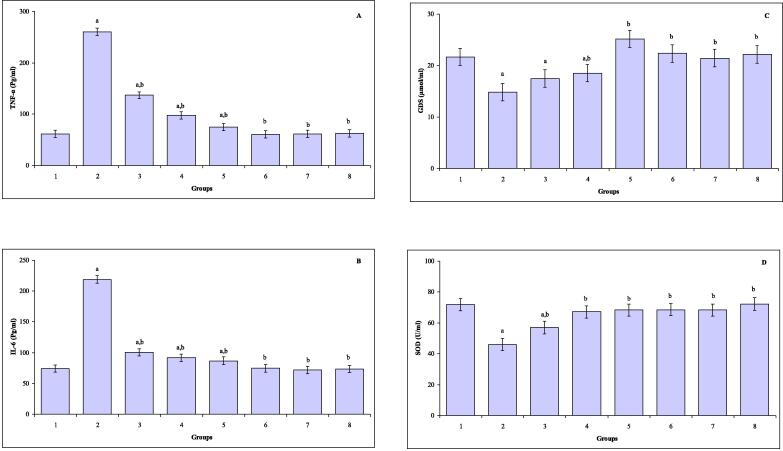
(**A–D**) The levels of serum TNF-α (A), IL-6 (B), GSH (C) and SOD (D) in control (group 1), TAA (group 2), CUR plus TAA (group 3), TQ plus TAA (group 4), CUR and TQ plus TAA (group 5), CUR (group 6), TQ (group 7) and CUR plus TQ (group 8) treated mice. ^a^Indicates a significant difference between group 1 and treated groups (2, 3, 4, 5, 6, 7 and 8). ^b^Indicates a significant difference between group 2, and groups 3, 4, 5, 6, 7 and 8.

The levels of serum TNF-α, IL-6, GSH and SOD are shown in Fig. 3A-D. In comparison with control mice, the levels of serum TNF-α were markedly increased in mice exposed to TAA (*P* ≤ 0.000), CUR plus TAA (*P* ≤ 0.000), TQ plus TAA (*P* ≤ 0.003) and CUR and TQ plus TAA (*P* ≤ 0.02). Statistically increases in the levels of serum IL-6 were noted in mice subjected to TAA (*P* ≤ 0.000), CUR plus TAA (*P* ≤ 0.01), TQ plus TAA (*P* ≤ 0.005) and CUR and TQ plus TAA (*P* ≤ 0.006). Noticeably decreases of serum GSH were observed in mice exposed to TAA (*P* ≤ 0.002), CUR plus TAA (*P* ≤ 0.002) and TQ plus TAA (*P* ≤ 0.04). The levels of serum SOD were decreased in mice exposed to TAA (*P* ≤ 0.000) and CUR plus TAA (*P* ≤ 0.000) compared to control mice.

## 4 Discussion

Environmental contamination by toxic chemicals is recognized as a global problem. Exposure to toxic substances is linked with serious health hazards. TAA causes harmful influences on the cellular and metabolic systems. This study is an attempt to elucidate the possible protective role of CUR and TQ against TAA induced hematological and biochemical alterations. The present study demonstrated that TAA induced serious changes in the hematological and biochemical parameters. Hematologically, the obtained results showed that the exposure to TAA caused statistical decreases in the values of RBC, Hb and Hct, while the value of WBC was significantly increased. Hematological characteristics have been widely used in the diagnosis of variety of diseases and pathologies induced by different toxicants, environmental pollutants and drugs in humans and animals (Yuan et al., 2014). The decrease in RBC value can be explained by the effect of TAA on the haematopoietic system which is susceptible to be damaged by exposure to TAA. Reduction in Hb concentration may be due to increased rate of breakdown of RBC and/or reduction in the rate of RBC formation. The present increase in WBC count due to TAA exposure may indicate an activation of the immune system in response to tissue damage caused by TAA.

The present data revealed that the exposure to TAA increased serum ALT, AST, ALP and TB levels. Liver AST, ALT, ALP and TB are frequently used as biomarkers of liver injury and diseases (Bozdağ and Eraslan, 2020, Datsko et al., 2020, Koyuncuoğlu et al., 2020, Li et al., 2020, Sun et al., 2021). Additionally, previous experimental studies showed that these parameters were significantly increased in animals exposed to TAA (El-Baz et al., 2019, Dwivedi et al., 2020, Gowifel et al., 2020, Bogahawaththa et al., 2021, El-Gendy et al., 2021, Younis et al., 2021).

The levels of serum TNF-α and IL-6 were statistically increased in rats exposed to TAA. TNF-α is a pleiotropic cytokine produced by a variety of immune cells including macrophages/monocytes. TNF-α can trigger multiple signaling pathways involved in inflammation, proliferation, and apoptosis (Yang and Seki, 2015). TNF signaling appears to be crucial in triggering liver inflammation, neutrophils and pro-inflammatory macrophage recruitment, as well as in activation of fibrogenic pathways that are central to the development of liver fibrosis (Wree et al., 2018). The inflammatory process that results from liver injury is characterized by the production of soluble mediators, including cytokines such as TNF-α and IL-1β derived from macrophages (Chen et al., 2012). Interleukins are immunomodulatory cytokines that mediate the communication between leukocytes or leukocytes and their target cells (Brocker et al., 2010). Interleukins are produced in large amounts during all kinds of immune responses, and the outcome of an inflammatory reaction is determined by the balance of cytokines produced (Brocker et al., 2010, Commins et al., 2010). IL-6 has long been recognized as an important proinflammatory cytokine whose expression is associated with many inflammatory disorders. Serum and intrahepatic levels of IL-6 are also strongly elevated in patients with acute and chronic liver diseases (Streetz et al., 2003). Previous studies showed that the levels of TNF-α and IL-6 were significantly increased in experimental animals exposed to TAA (Abdul-Hamid et al., 2017, Ali et al., 2021, Elnfarawy et al., 2021, Gowifel et al., 2020, Gregolin et al., 2021, Han et al., 2021, Ibrahim et al., 2018).

The present study showed that TAA induced oxidative stress as indicated by significant decline of serum GSH and SOD levels. Oxidative stress is defined as excess production of reactive oxygen species (ROS) relative to antioxidant defense. Oxidative stress has been shown to participate in a wide range of diseases. However, previous experimental investigations revealed that TAA induced oxidative stress which confirmed by significant alterations of oxidative markers such as GSH and SOD (Sukalingam et al., 2018, Hafez et al., 2019, Bashandy et al., 2020, Đurašević et al., 2021).

The results of the present study showed that CUR and TQ treatment significantly attenuated the changes of hematological (RBC, Hb, Hct and WBC) and biochemical (ALT, AST, ALP, TB, TNF-α, IL-6, GSH and SOD) parameters induced by exposure to TAA. Moreover, the most attenuation was observed in mice treated with CUR and TQ. From the present new findings, the possible mechanism of CUR and TQ attributed to their antioxidant activities which evaluated by GSH and SOD levels. The desirable preventive or putative therapeutic properties of CUR have also been considered to be associated with its antioxidant and anti-inflammatory properties. However, CUR has been investigated extensively as an anti-cancer, anti-aging and wound healing agent (Agrawal and Mishra, 2010, Lima et al., 2011, Akbik et al., 2014;). Abo-Zaid et al. (2020) evaluated the regulatory immune effect of CUR in hepatic cirrhosis induced by carbon tetrachloride (CCl_4_) in rats. The results revealed that CUR inhibited hepatic fibrosis and liver fibrogenesis with regulation of the immune system mechanism against CCl_4_ toxicity. Abubakar et al. (2020) evaluated the ameliorative effects of CUR on lead (Pb)-induced hepatorenal toxicity in rats. The results showed that CUR attenuates Pb-induced hepatorenal toxicity via chelating activity and inhibition of oxidative stress. Li et al. (2021) studied the effect of CUR on liver damage induced by radiation in rats.

CUR inhibited the liver damage by reducing the levels of ALT, AST, ALP, LDH and maleicdialdehyde (MDA), while the levels of GSH, SOD and caspase activated DNase (CAD) were significantly increased. Moreover, CUR showed anti-apoptosis and anti-inflammation properties and inhibited the NF-κB pathway resulting in the protection of the liver from damage induced by radiation. Concerning the vital roles of TQ, several investigations have explored the development of TQ analog compounds with notable efficacy for different types of diseases (Goyal et al., 2017). Previous studies explained that TQ induces an antioxidant, anti-inflammatory, anti-apoptotic and immunomodulatory effects (Aycan et al., 2014, Shaterzadeh-Yazdi et al., 2018, Abdel-Daim et al., 2019, Guo et al., 2020, Akgül et al., 2021, Landucci et al., 2021, Khalifa et al., 2021). Al Aboud et al. (2021) investigated the hepato-protective influence of TQ in rats exposed to arsenic (As). Hepatic oxidative damage induced by As were detected by the decline of antioxidant markers [SOD, GSH, catalase (CAT), glutathione peroxidase (GPx) and glutathione reductase (GR)], while the level of with MDA was statistically increased. Additionally, ALT, AST, ALP and TB levels, apoptotic marker, liver fibrotic markers, TNF-α, IL-6 and myeloperoxidase (MPO) were significantly increased due to As exposure. The results showed that TQ attenuated these alterations which attributed to its antioxidant, anti-inflammatory, anti-apoptotic, and fibrolytic properties.

Based on the present study, it can be concluded that above mentioned results confirmed the usefulness of CUR, TQ and their combination against TAA toxicity. Further experimental investigations are required to establish the efficacy of different concentrations and doses of CUR and TQ as potential natural preventive agents against TAA toxicity, other toxic factors and pathogens.

## Declaration of Competing Interest

The authors declare that they have no known competing financial interests or personal relationships that could have appeared to influence the work reported in this paper.
